# No Sedation, No Traction, and No Need for Assistance: Analysis of New Prakash's Method of Shoulder Reduction

**DOI:** 10.1155/2020/4379016

**Published:** 2020-01-04

**Authors:** Tolgahan Kuru, Haci Ali Olcar, Ali Bilge, Gurdal Nusran, Recai Ozkilic, Canan Akman, Lakshmanan Prakash

**Affiliations:** ^1^Department of Orthopedics and Traumatology, Onsekiz Mart University Medical Faculty, Canakkale, Turkey; ^2^Department of Orthopedics and Traumatology, Turkish Ministry of Health Yozgat City Hospital, Yozgat, Turkey; ^3^Department of Orthopedics and Traumatology, Turkish Ministry of Health Düzce State Hospital, Düzce, Turkey; ^4^Department of Emergency Medicine, Onsekiz Mart University Medical Faculty, Canakkale, Turkey; ^5^Institute for Special Orthopedics, Chennai, Tamil Nadu, India

## Abstract

**Materials and Methods:**

A total of 19 patients who were admitted to the emergency department with the diagnosis of anterior shoulder dislocation participated in this study. The diagnosis of shoulder dislocation was established in the emergency department with physical examination and anteroposterior shoulder radiography. The method was applied only once to the patients in the sitting position by the same physician without using any help, traction, anesthesia, analgesia, and myorelaxant.

**Results:**

The mean age of the patients was 37.3 ± 13.1 years. Among them, 36.8% (*n*=7) were female and 63.2% (*n*=12) were male. Recurrent dislocations were observed in 21.1% (*n*=4) of the patients. The success rate of the method was 94.7% (*n*=18). No complication was noted in the patients. The mean procedure time was 243 ± 38 seconds.

**Conclusion:**

Prakash's method is a safe method for anterior shoulder dislocations that can be quickly performed with no need for sedation, assistance, and traction and has a high success rate.

## 1. Introduction

Being one of the most mobile joints of the body, the glenohumeral joint is prone to dislocations due to its high joint mobility. It has been reported that the incidence of glenohumeral joint dislocation in the general population is 2–8% [1, 2]. In the developed countries, the prevalence of glenohumeral dislocation is reportedly 21.9–23.9/100.000, with the majority reported as anterior [3, 4]. Falling and sports injuries are among the common causes of glenohumeral dislocations that affect young men more often [4].

More than 20 methods have been suggested for the treatment of glenohumeral joint dislocations, which are often treated conservatively [5, 6]. The success rate and complications vary according to the method used [7]. Although most anterior dislocations can be reduced at emergency departments in most hospitals, certain dislocations may require multiple methods. Reduction is rarely performed with surgery under anesthesia [8, 9].

The optimal method of reduction can be described as one which requires minimum assistance, is highly effective, is quickly performed with minimum pain, is safe, or has few complications [8]. On the other hand, research shows that the widely used methods such as the Hippocratic method or Kocher methods fail to yield the desired results or have a high complication rate [7]. Therefore, there are still ongoing attempts to find an optimal method of reduction.

The new method of reduction recently developed by Prakash [10] has revealed that anterior shoulder dislocations can be quickly reduced without pain or sedation and with a high success rate. Accordingly, this study aims to evaluate the effectiveness and safety of the method of reduction developed by Prakash.

## 2. Materials and Methods

It was found in screening of the hospital records that 25 patients presented to the emergency department of our hospital with anterior shoulder dislocation between January 2019 and April 2019. Among these, a total of 19 patients treated with Prakash's method for shoulder dislocation whose complete data could be accessed were included in the study. Patients with concomitant fractures, who presented more than 24 hours after the trauma, patients with a neurovascular trauma or any condition that prevented placing them in the sitting position, patients with multiple traumas, and those treated with other methods were excluded from the study. Anterior shoulder dislocations were assessed based on physical examination and anteroposterior radiography. Following this, control radiographs were taken to evaluate the reduction. Success of the reduction was confirmed with anteroposterior radiographs and physical examination. Neuromuscular examination was then performed.

Patients' age, gender, trauma mechanism, side of the shoulder dislocation, presence of a history of previous shoulder dislocation, reduction success, and postreduction complications were examined from the hospital records.

The patients were informed about the study, and the study was performed with those who volunteered to participate. The sociodemographic data, information on the side of the dislocation (left or right), and history of dislocation were collected from the patients. While the reduction method was applied, complications, if any, success rate, and reduction time were evaluated. No sedative or myorelaxant was used prior to the procedure, and no traction was applied during the procedure.

**Figure 1:**
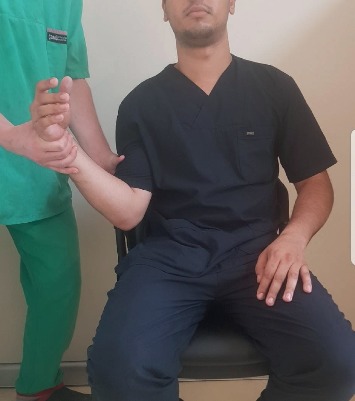
External rotation until the side dislocated is fully externally rotated.

**Figure 2:**
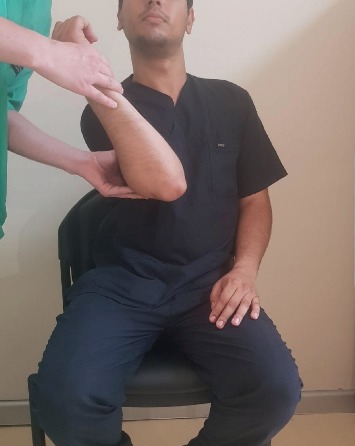
Shoulder adduction when the limb of the patient is in external rotation at the 2- or 3-o'clock direction.

**Figure 3:**
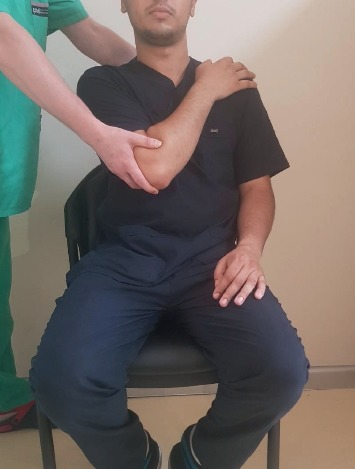
The limb is internally rotated to ensure that the hand of the patient can touch the opposite shoulder on the dislocated side.

As defined above, Prakash's method was applied while the patients were in the sitting position with a fixed scapula [10]. Reduction was performed once by the same physician without any assistance. No sedative or myorelaxant was used prior to the procedure, and no traction was applied during the procedure. The steps followed are as follows:

- External rotation is gently carried out until the side dislocated is fully externally rotated, with no attempts at abduction or adduction (∼1 min) (Figure 1)
- Shoulder adduction is done when the limb of the patient is in external rotation at the 2- or 3-o'clock direction (Figure 2)
- The limb is internally rotated to ensure that the hand of the patient can touch the opposite shoulder on the dislocated side (Figure 3)

The patients whose dislocations could not be reduced with Prakash's method were operated using the Hippocratic method.

This study was performed in accordance with the Declaration of Helsinki, based on the approval of the Ethics Committee of Yozgat City Hospital, Turkish Ministry of Health, with 13/03/2019 dated and 2017-KAEK-189_2019.03.13_09 numbered decision.

## 3. Results

This study was performed based on the data of 19 patients. The mean age of the patients was 37.3 ± 13.1 years. Among them, 36.8% (*n*=7) were female and 63.2% (*n*=12) were male. While dislocation occurred on the left shoulder in 31.6% of the patients (*n*=6), it was in the right shoulder in 68.4% of them (*n*=13). 78.9% of the patients (*n*=15) presented themselves with a first-time shoulder dislocation. Demographic and clinical features of the patients are given in Table 1. No fracture concomitant with shoulder dislocation was noted in patients. No sedation or traction was applied to patients during reduction. The success rate of the method was 94.7% (*n*=18). Reduction could not be performed in only one patient, who was then sedated and operated using the Hippocratic method. The mean reduction time was 243 ± 38 seconds.

## 4. Discussion

Glenohumeral joint is the joint where dislocation is most common, since the large humeral head is jointed onto the relatively small shallow glenoid fossa and movable in all directions [11]. For the first time, a conservative method for the reduction of shoulder dislocations was found by Hippocrates [12]. The Hippocratic method, which was widely preferred as it is a historical method, is not commonly used today due to its association with complications [13]. More than 20 methods have been suggested for the reduction of shoulder dislocation [14]; however, none of them offers optimal success (Table 2). There is no consensus on the optimal method of reduction, but it should be one that is easy to use, does not require help, requires no sedation or traction, has a high success rate and low complication rate, and is quickly applied. Yet, the reported methods of reduction fail to meet optimal requirements.

Prakash's method was first applied in 2016 since when it has been successfully used for the reduction of anterior shoulder dislocations in different regions, and in 2018, it was reported that it was used without any complication in 147 patients with a 100% success rate [10]. Amongst its major advantages are a high success rate, few or no complications, quick application, no need for sedation or traction, and no need for assistance.

Scapular manipulation [17], traction countertraction [18], and Chair [5] methods have a high success rate relative to other methods for shoulder reduction. The Chair method requires active participation of the patient, while the dislocations of almost all patients could be successfully reduced in the studies by Güler et al. [5] and Chung et al. [21]. Previous studies reported a lower success rate for reduction with a similar Chair method [22, 23]. On the other hand, traction poses a risk for neurovascular injury. Baykal et al. [17] reported a high success rate for the scapular manipulation method; yet, sedatives were needed for some patients, and attaching 3–7 kg weights to the affected arm was recommended for the methods using traction. In contrast, Adhikari et al. [24] reported a lower success rate for scapular manipulation. In relation to the traction countertraction method, Ghane et al. [18] stated that the success rate in 50 cases was 73% in the first attempt and 100% in the second attempt, but sedatives were used in all TCT cases. The authors further reported that the success rate of TCT was lower in the first attempt in comparison with the modified scapular method and reduction took somewhat longer time than Prakash's method. The systematic review recently prepared by Alkaduhimi et al. [25] found that the scapular manipulation method is the fastest method with 1.75 min, followed by “Fast, Reliable, and Safe” (FARES) with 2.24 min and the traction countertraction method with 6.05 min. This review does not include Prakash's method, but it can be argued that Prakash's method may yield more successful results and offer some advantages such as low complication rate, no need for assistance, and short reduction time. More extensive studies may provide more comprehensive information by comparing the successful methods for reduction in anterior shoulder dislocations, including Prakash's method.

A relatively small number of patients may be considered a limitation of this study. However, given that Prakash's method is a novel technique used for shoulder dislocations, further studies will probably include a larger number of participants over time.

In conclusion, Prakash's method offers a high success rate in the acute conservative treatment of anterior shoulder dislocations. The importance of Prakash's method becomes clear considering the strengths of this method such as quick application, no need for help, few or no complications, no use of traction associated with complications, and no need for sedatives or similar medications as the patients experience little pain during the application. As such, orthopedic surgeons and emergency physicians can consider this method as an ideal method to use to treat acute shoulder dislocations at emergency departments.

## Figures and Tables

**Table 1:** Demographic, clinical, and reduction-related data.

Demographic and clinical data		
Age (year)	Mean ± SS	37.3 ± 13.1
Sex	*N* (%)	
Female		7 (36.8)
Male		12 (63.2)
Lesion side	*N* (%)	
Right		13 (68.4)
Left		6 (31.6)
Shoulder dislocation	*N* (%)	
First-time		15 (78.9)
Recurrent		4 (21.1)

*Reduction-related data*		
Fracture after reduction	*N* (%)	0
Sedation (+)	*N* (%)	0
Traction (+)	*N* (%)	0
Reduction success rate	*N* (%)	18 (94.7)
Reduction time (sec)	Mean ± SS	243 ± 38

**Table 2:** Reduction methods used in anterior dislocations.

Author-year	Reduction method	Number of patients	Sedation	Acute complications	Reduction time	Success rate (%)
Marinelli and de Palma, 2009 [15]	External rotation	31	6/31	5/31	3 min	89
Chitgopkar and Khan, 2005 [16]	Kocher	12	0	—	—	83
Baykal et al., 2004 [17]	Scapular manipulation	41	4/41	0	—	90.2–100
Ghane et al., 2014 [18]	Traction countertraction	50	50/50	0	470 ± 227 sec	73–100
Ghane et al., 2014 [18]	Modified scapular manipulation	47	0	0	79.35 ± 82.49 sec	89–97
Güler et al., 2015 [5]	Chair	47	0	0	3.0 ± 1.2 min	97.8
Güler et al., 2015 [5]	Matsen	27	0	0	4.7 ± 2.3 min	92.5
Singh et al., 2012 [19]	Modified Milch	31	31/31	4/31	—	83.9
Maity et al., 2012 [20]	FARES	80	80/80	0	2.16 ± 0.96 min	95
Sayegh et al., 2009 [7]	Hippocratic	51	0	0	5.55 ± 1.58 min	72.5

## Data Availability

The data used to support the findings of this study are included within the article.
